# Sexual Health in Atopic Dermatitis: Impact of Skin Clinical Control

**DOI:** 10.1002/clt2.70115

**Published:** 2025-11-08

**Authors:** Jorge Sánchez, Gabriel Montoya, Ana Caraballo, María‐Fernanda Ordoñez‐Rubiano, Margarita Velasquez, Claudia Arenas, Julián Londoño, Elizabeth García

**Affiliations:** ^1^ Group of Clinical and Experimental Allergy Hospital “Alma Mater de Antioquia” University of Antioquia Medellín Colombia; ^2^ Faculty of Medicine University of Antioquia Medellín Colombia; ^3^ Internal Medicine Division Dermatology Hospital Militar Central Cayre Institution Bogotá Colombia; ^4^ “Centro de Investigaciones Dermatológicas” Group University of Antioquia Medellín Colombia; ^5^ “Centro Dermatológico Federico Lleras Acosta, E.S.E” University Hospital Bogotá Colombia; ^6^ Allergology Unit Medical Surgical Unit of Otorhinolaryngology (UNIMEQ‐ORL) Bogotá Colombia

**Keywords:** anxiety, atopic dermatitis, depression, sex, treatment

## Abstract

**Background:**

Sexual health in patients with atopic dermatitis (AD) remains scarcely studied. Identifying the problematic of sexual health disorders (SHD) in AD and associated factors is important for the design and implementation of targeted interventions.

**Objective:**

To describe the frequency of sexual health disorders (SHD) in AD patients, identify risk factors and assess whether improved AD control with pharmacotherapy is associated with changes in SHD.

**Methods:**

We performed a prospective observational study in AD patients over 18 years of age. Participants completed a survey assessing clinical aspects of SHD and AD at baseline and 5–7 months after initiating specialists‐recommended treatment. For AD severity evaluation we used SCORAD and POEM scales and for SHD we used SyDSF‐AP, IFSF, and MGH‐SFQ.

**Results:**

A total of 221 AD patients were enrolled. At baseline, the frequency of SHD varied according to AD severity (SHD in severe AD 100%, in moderate AD 96%, and in mild AD 56%). Risk factors for SHD were AD severity (SCORAD OR 3.88 [95% CI 2.68–4.73], POEM OR 4.67 [95% CI 3.05–5.79]), skin area affected (OR 3.15 [95% CI 2.88–5.19]), and disease duration (OR 3.75 [95% CI 1.88–4.91]). Improved AD control through pharmacotherapy reduced SHD frequency in mild AD (relative reduction [RR]: −60%), moderate AD (RR: −41%), and severe AD (RR: −28%).

**Conclusion:**

Atopic dermatitis was frequently associated with SHD even in mild forms of the disease. However, AD clinical control reduced the frequency of SHD, and consequently improving the quality of life of patients.

## 1 Introduction

Atopic dermatitis (AD) is one of the most common chronic skin diseases affecting 3%–15% of the adult population [1, 2, 3, 4]. While AD often begins in childhood and most patients experience remission before puberty, the most severe forms can persist into adulthood, significantly impacting quality of life [5, 6, 7]. The chronic nature of the disease, enhanced by the visibility of the skin lesions, is associated with psychological complications such as alexithymia, anxiety, and depression [5, 6, 7]. Many patients avoid social contact, which in turn generates difficulties in their academic, work, family development, and in the development of social relationships [5, 6, 7].

Sexual health must be approached from a holistic perspective [8], as it can be affected by multiple factors. Sexual health disorders (SHD) in the general population appear to be under‐diagnosed due to social and cultural pressures that keep the subject taboo [9]. Some studies suggest that between 5% and 50% of the sexually active population experience some type of SHD per year and this could be higher in patients with chronic diseases [8, 10].

Due to the emotional and mental health vulnerability of AD patients [5, 6, 7], they represent a population at increased risk for SHD [11]. Additionally, AD common symptoms like eczema and pruritus, may interfere directly with sexual activity. This is an aspect that has been scarcely explored; a systematic review published in 2021 [12], identified 82 articles related to the topic but only five studies evaluated the impact of AD on SHD. The review concluded that the available information to evaluate the relationship between these two conditions is limited despite their mutual occurrence seems to be frequent in the adult population (40%–60%). Some additional studies have evaluated the impact of SHD in AD [13, 14, 15]; however, these studies rely on extrapolation from other diseases and author assumptions, rather than AD disease specific data.

Therefore, this knowledge gap may have important impacts on the management of AD. Identifying risk factors between SHD and AD is important for the design and implementation of effective interventions. Considering this scenario, our objective was to describe the frequency of SHD in AD patients, identify potential risk factors, and evaluate if improved AD clinical control with pharmacotherapy could result in a reduction of SHD.

## 2 Methods

### 2.1 Type of Study and Population

We conducted a prospective observational study involving six health centers in two Colombian cities. Considering the average age of sexual activity in the Colombian population (18 years) (https://www.dnp.gov.co), we invited adult AD patients (≥ 18–50 years of age) who assisted for the first consultation at any of the study health centers to participate in the study. The diagnosis of atopic dermatitis was made according to U.K. criteria [16, 17] by AD specialist (allergist or dermatologist) according to; controller treatment was selected according to each physician criteria based in international recommendations [18]. Most patients were included during their first appointment with an AD specialist in participating centers; during the first appointment most patients were not receiving a controller treatment according to international guidelines (67%), so the number of patients who received systemic therapy was relatively low since local regulations require a step‐by‐step management before using systemic therapies and the availability of the necessary paraclinical tests to request them. Patients with other skin conditions (e.g., psoriasis, or other chronic skin comorbidities) or patients with SHD clearly attributable to another cause were excluded. All patients who agreed to participate were given a survey at the beginning of the study and 5–7 months after the treatment recommended by their AD specialist (allergist or dermatologist). The questionnaire was designed by sexual health specialists, allergists, and dermatologists.

### 2.2 Atopic Dermatitis Severity

AD severity was evaluated before and after pharmacotherapy according to SCORAD (SCOre Atopic Dermatitis) scale and the POEM (Patient Oriented Eczema Measure) scale. Patients with AD were classified according to the SCORAD into “severe” (> 45 points), “moderate” (26–44 points), “mild” (< 25 points). POEM was used as a continuous variable. Where appropriate we use sub analysis according to severity classification: “clear/almost clear” 0–2 points, “mild” 3–7 points, “moderate” 8–16 points, “severe” 17–24 points, “very severe” 25–28 points.

### 2.3 Description of Sexual Function

To evaluate sexual health, we conducted a questionnaire including three standardized and validated international scales; SyDSF‐AP (“Female Sexual Health and Dysfunctions in Primary Care”) [19], IFSF (Female Sexual Function Index) [20], and MGH‐SFQ (Massachusetts General Hospital‐Sexual Functioning Questionnaire) [21]. Briefly. The three questionnaire assesses “Yes or no” the presence of some SHD. The SyDSF‐AP is a validated 21‐item questionnaire and evaluated pain during sexual intercourse, low libido or little interest in sex, and problems with arousal or orgasm. IFSF consists of 19‐item and assesses SHD such as sexual desire, arousal, stimulation, orgasm, satisfaction, and pain. MGH‐SFQ consists of five‐items and evaluated sexual functioning, including interest, arousal, orgasm, and satisfaction. We previously carried out a pilot test to evaluate the cultural adaptability and consistency of the survey, which allowed us to adapt the questions to some linguistic expressions of the country. We also included some specific questions about how dermatitis impacts sexual activity.

Using these scales, we explored three domains of sexual health: initiating and maintaining a relationship (“relationship”), feelings about sex and sexual stimulation (“Sexual desire”), initiation, enjoyment, and frequency of sexual intercourse (“Sexual activity”). The survey was completed virtually by each patient using a numerical code to ensure anonymity.

### 2.4 Statistical Analysis and Sample Size

Statistical analyses were conducted with GraphPad Prism 9, JAMOVI (Sidney, Australia), and SPSS 26 (IBM Corporation, Armonk, NY). Each center was required to include at least 25 patients to perform a stratified comparison according to AD severity. For descriptive characterization, median, confidence interval, and percentages, were used.

Comparisons between AD severity groups (mild, moderate, severe), and SHD (with or without SHD) groups, were performed using the Kruskal–Wallis test for multi‐groups comparisons and the Mann–Whitney test for “before and after” comparisons. A “*p*” value ≤ 0.05 was considered statistically significant. Treatment response was assessed according to the net reduction in SHD (baseline frequency–final frequency) and the relative reduction in SHD ((100% × SHD final frequency/SHD baseline frequency)–100).

The survey comprised three sections: sociodemographic data, AD data and SHD data. Patients who agreed to participate in the study but did not complete all three sections were excluded from the analyses.

### 2.5 Ethical Considerations

This study was approved by the ethics committee of the University of Antioquia (Medellín, Colombia) (Code F‐017‐00). All participants provided a written informed consent.

## 3 Results

### 3.1 Characteristics of the Study Population

A total of 263 patients agreed to participate. Forty‐two of these did not complete the three sections of the survey specially the SHD data. When comparing participants who completed SHD data and those who did not, no significant differences were found in AD severity. Mental diseases (e.g., anxiety, depression) based in clinical history were frequent (Table 1). Nevertheless, non‐responders of SHD data had a higher frequency of men (55% vs. 39.8%) and were older (median age 48 years, 95% CI 40–60) compared with responders (median age 28 years, 95% CI 18–48).

**TABLE 1:** Sociodemographic and clinical characteristics of the patients.

Variable	221 patients
Sex	Male 88 (39.8%)
	Female 133 (60.1%)
Gender	Male 89 (40.3%)
	Female 132 (59.7%)
Sexual orientation	Heterosexual 213 (96.4%)
	LGTBI 8 (3.6%)
Age	28 years (CI 18–48)
Men	29 years (CI 20–46)
Women	27 years (CI 18–48)
Asthma	69 (31.2%)
Rhinitis	146 (66%)
Conjunctivitis	101 (45.7%)
Mental health	
Mental diseases (except anxiety and depression)	48 (21.7%)
Anxiety and/or depression	128 (57.9%)
AD severity	
SCORAD	Median 34 (CI 18–64)
POEM	Median 14 (CI 6–28)
Skin area affected	
Face	21 (9.5%)
Hands	41 (18.5%)
Genital areas	39 (17.6%)
Baseline pharmacotherapy	
Topical steroids	212 (94.9%)
Hydration	123 (55.6%)
Steroid cycle	23 (10.4%)
Immunosuppressants	12 (5.4%)
Phototherapy	4 (1.8%)
Dupilumab	2 (0.9%)

Abbreviations: AD; Atopic dermatitis. CI: Confidence Interval. LGTBI: Lesbian, Gay, Transgender, Bisexual, Indefinite.

A total of 221 patients were included in the analysis. There was a predominance of female participants (Table 1). The presence of eczema in genitals, hands, or face was more frequent among patients with SCORAD greater than 25 points (*p* < 0.03).

### 3.2 Sexual Health Disorders and Risk Factors

Overall, 188 patients (85%) had a SHD in at least one of the three domains evaluated, and 57.4% had a SHD in at least two domains. AD patients with moderate or severe symptoms according to SCORAD had a higher frequency of SHD than those with mild (Table 2).

**TABLE 2:** Association between Atopic dermatitis (AD) severity and Sexual Health Disorder (SHD).

Variable	Global (221)	AD mild (*n* 68)	AD moderate (*n* 112)	AD severe (*n* 41)
AD patient with SHD	188 (85%)	39 (56.5%)	108 (96.4%)	41 (100%)
SHD “relationship” domain	134 (60.6%)	26 (38.2%)	70 (62.5%)	38 (92.6%)
SHD “sexual desire” domain	121 (54.7%)	26 (38.2%)	60 (53.5%)	35 (85.3%)
SHD “sexual activity” domain	93 (42%)	13 (19.1%)	42 (37.5%)	38 (92.6%)

*Note:* Severity of AD was defined according to SCORAD (Mild < 25 points, Moderate 26 to 50 points, severe > 51 points). SHD (Sexual health disease) was defined according to the domains of the questionnaires.

There were some differences in the affected SHD domains according to biological sex. Male patients had a greater impact in the relationship domain (difficulty initiating a relationship), while female patients were more affected in the sexual desire and sexual activities domains (reporting unpleasant sexual activities, negative emotional impact, and physical pain during sexual activities).

**FIGURE 1:**
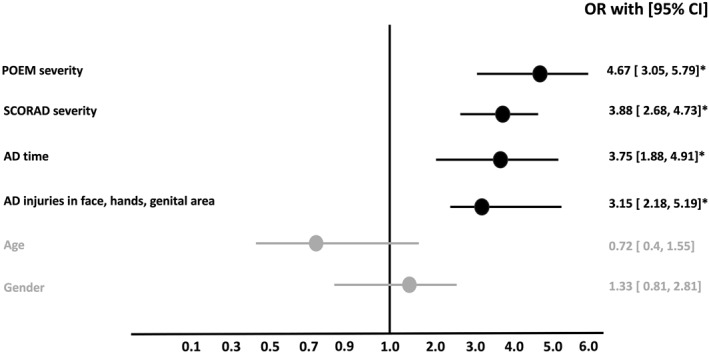
Six variables were evaluated in AD patients and four were associated with SHD. AD time: number of years with AD. * = *p* < 0.05.

Risk factors for presenting SHD were related to the location of the skin eczema (face, hands, genital area), the AD severity, and longer disease duration (Figure 1). Other factors such as age or sex (male, female, other) did not have a significant association with SHD. Mental illnesses such as anxiety and depression were associated with an increased risk of SHD, however, when these variables were adjusted for AD severity, the association was not maintained.

### 3.3 Control of Atopic Dermatitis and Impact on Sexual Health Disorders

**FIGURE 2:**
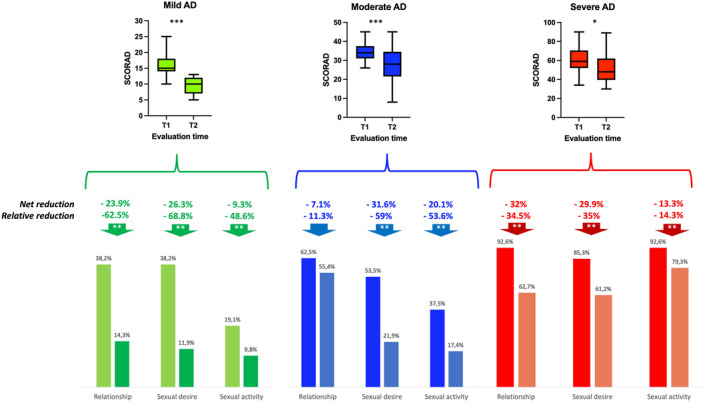
Change in atopic dermatitis severity was associated with a reduction in sexual health disorders. T1 first visit to AD specialist. T2 second visit after 4–8 months * = *p* < 0.05, ** = *p* < 0.01, *** = *p* < 0.001.

Following pharmacotherapy, a reduction in AD severity was observed (Figure 2). This improvement was associated with a reduction in the SHD frequency. The greatest improvement was observed in the relationship and sexual desire domains in patients with mild AD, while the largest impact in the sexual activity domain was observed in those with moderate AD. The overall relative reduction in SHD according to AD severity was mild −60%, moderate −41%, severe −28%. Relative reduction was also important independent of AD severity (Global relative reduction for mild −59.9%, moderate −41.3%, severe −28.2%). After pharmacotherapy, the frequency of anxiety and depression presented a reduction (Baseline 57.9% vs. after 28% *p* = 0.01) but after adjusted the regression model for these diseases or other mental illness the reduction of SHD did not presented a significant change indicating that pharmacotherapy effect on SHD was not depended of the effect in mental illness.

As an exploratory analysis, some questionnaire questions allow us to establish the impact of SHD on quality of life and sexual health and we did a correlation analysis of these variables with the SCORAD (baseline and after pharmacotherapy); SCORAD and SHD quality of life presented a significant correlation (baseline R 0.814 *p* 0.001 after pharmacotherapy R 0.916 *p* < 0.001).

## 4 Discussion

For health professionals who are not specifically trained in sexual health, addressing this topic can be challenging due to the emotional, cultural, and social factors that make such conversation difficult [22, 23]. Currently there are several structured clinical questionnaires to evaluate the presence of SHD [19, 20, 21, 24, 25] and anonymity is often preferred when possible as many patients feel uncomfortable or judged when discussing sexual health issues [26]. These considerations highlight the need for a sensitive and structured approach when evaluating SHD. However, independent of the strategy, SHD assessment must be carried out actively in clinical appointments, mainly in populations at risk, such as patients with chronic diseases [22, 27, 28, 29].

Our study provides some interesting findings:

1. SHD occurred in over 50% of AD patients, even among those with mild skin symptoms.
2. SHD were associated with AD severity, the skin area affected, and the time with AD.
3. Differences in SHD affected domains were observed according to biological sex.
4. AD control with pharmacotherapy reduces SHD.

SHD encompasses diverse conditions with a common point: no satisfactory sexual relations. Given the emotional and psychological burden of AD [5, 6, 7], these patients are at high risk for SHD. There is a limited evaluation of the relationship between AD and SHD [12, 30, 31, 32, 33, 34], but their mutual presence seems to be frequent, and our results support that SHD is common in this population specially when severe skin symptoms are present. Unfortunately, sexual health is rarely assessed in clinical practice, in our study more than 98% of the participants mentioned that this was the first time they had been asked about this topic.

Recognizing the problem and risk factors allows the development of possible targeted interventions. We observed that improved clinical control of AD with pharmacotherapy was associated with a significant reduction in SHD frequency. Notably, even if the patients did not achieve complete clinical control, partial skin improvement was still associated with a reduction in SHD.

We also found a more frequent refusal to complete the SHD survey among older and male patients. Additionally, we observed differences in SHD affected domains according to biological sex. This may be secondary to sociocultural patterns [35, 36, 37]. In Latin America, older adults are less likely to discuss about their sexuality and men are often culturally expected to initiate a relationship, potentially impacting personal, social and professional outcomes.

Our study presents some strengths and weaknesses. To our knowledge, this is the first study to show that SHD frequency can be reduced among AD patients with pharmacotherapy control. The multicenter design and the inclusion of validated, internationally recognized sexual health scales (adapted to the Colombian cultural context) enhance the reliability and applicability of our findings. Additionally, evaluating patients both before and after treatment provided insight into the potential reversibility of SHD with clinical improvement of AD.

However, there are also some limitations. The self‐reported questionnaires may generate reporting bias, particularly in a culturally sensitive topic such as sexual health. The significant lower proportion rate of response of men and older adults with AD leaves a knowledge gap in this population. Nevertheless, non‐response in these patient groups highlights specific barriers to SHD assessment and management that need to be addressed. Although we observed similar results using two scales (SCORAD, POEM), with CROM (clinician‐reported outcome measure) and PROM (patient‐reported outcome measure) characteristics, it is necessary to conduct studies with other scales for AD (e.g., EASI, NRI) which prioritize different domains of AD to define severity.

Furthermore, while our study shows an association between improved AD control and reduced SHD, causality cannot be established due to the observational design. Future longitudinal and interventional studies are needed to confirm these findings and explore the mechanisms underlying the relationship between AD and sexual health.

## 5 Conclusions

According with our results, atopic dermatitis negatively impacts sexual health even in its mild forms. However, adequate control of risk factors and effective disease management can reduce SHD and consequently improve the quality of life of patients. These findings support the need for a holistic approach to the management of individuals with AD.

## Author Contributions

**Jorge Sánchez:** conceptualization, investigation, methodology, funding acquisition, writing – original draft. **Gabriel Montoya:** conceptualization. **Ana Caraballo:** conceptualization, methodology, writing – original draft. **María‐Fernanda Ordoñez‐Rubiano:** conceptualization. **Margarita Velasquez:** conceptualization. **Claudia Arenas:** conceptualization. **Julián Londoño:** methodology. **Elizabeth García:** conceptualization.

## Funding

This article is the result of an initiative funded by the Colombian Association of Allergy, Asthma and Immunology (ACAAI). The ACAAI is a non‐profit association funded by multiple laboratories. For this initiative, the ACAAI received financial support from the Pfizer and Sanofi laboratories. The entities that funded the ACAAI did not intervene in the writing, direction or execution of this manuscript.

## Conflicts of Interest

The authors declare no conflicts of interest.

## Supporting information


Supporting Information S1

## Data Availability

The data that support the findings of this study are available from the corresponding author upon reasonable request.
