# Supplementary material for: Sexual Health in Atopic Dermatitis: Impact of Skin Clinical Control
Source: Clin Transl Allergy. 2025 Nov 8;15(11):e70115. doi: 10.1002/clt2.70115 (PMC12596026; doi:10.1002/clt2.70115)
Supplement: Supplementary file 1 — Supporting Information S1 [file CLT2-15-e70115-s001.docx]

| **GENERAL DATA** | | | | | |
| --- | --- | --- | --- | --- | --- |
| DATE: | | AGE: | | | SEX: |
| CITY OF RESIDENCE: | |  | | |  |
| Marital Status: Married. Single. Separated. Widowed. Divorced. | | | | | |
| In the last 6 months, have you had or do you have a stable partner?: No Yes. | | | | | |
| Children? No Yes. | | | | How many? | |
| Level of education: Elementary. High school. Higher education. | | | | | |
| Employment status: Active. Unemployed. Retired. | | | | Smokes: No Yes. | |
| Alcohol: No Yes. | | Regular exercise: No Yes. | | | Recreational drugs: No Yes. |
| **COMORBIDITIES: Has a doctor diagnosed you with…?** | | | | | |
| Asthma: No Yes. | | Conjunctivitis: No Yes. | | | Rhinitis: No Yes. |
| Other: No Yes. | | Keratoconjunctivitis: No Yes. | | | Anxiety: pts |
| Depression: pts | | If yes, which one(s)? | | | |
|  | |  | | |  |
| **DERMATITIS DATA (first consultation)** | | | | | |
| SCORAD: pts . | | POEM: pts . | | | DLQI: pts . |
| Age of onset (years or months) | | | | |  |
| **TREATMENT (first consultation)** | | | | | |
| AntiH1 1st generation: No Yes. | | | AntiH1 2nd generation: No Yes. | | |
| Topical steroid: No Yes. | | | If yes, regarding potency: Low. Moderate. Severe. | | |
| Tacrolimus : No Yes. | | | Oral steroid; Received, Received, Never | | |
| Cyclosporine: Received, Receives, Never | | | | Mycophenolate: Received, Receives, Never | |
| Methotrexate : Received, Receives, Never | | | | Azathioprine: Received, Receives, Never | |
| Other treatment: No Yes. | | | | Which? | |
| **Sexual health and dysfunction** | | | | | |
| Are you satisfied with your sex life? Not at all. A little. Somewhat. Quite a bit. A lot. Extremely. | | | | | |
| Has your interest in sex waned? Not at all. A little. Somewhat. Quite a bit. A lot. Extremely. | | | | | |
| Do you experience fewer pleasurable sensations during sexual intercourse?  Never. Almost never. Sometimes. Often. Almost always. Always. | | | | | |
| Do you feel excited or stimulated during sexual intercourse?  Nothing. A little. Some. Quite a bit. A lot. Extremely. | | | | | |
| Do you feel excited or stimulated during sexual intercourse?  Nothing. A little. Some. Quite a bit. A lot. Extremely. | | | | | |
| Do you feel pain during sexual intercourse?  Nothing. A little. Some. Quite a bit. A lot. Extremely. | | | | | |
| Do you have orgasms? Never. Almost never. Sometimes. Often. Almost always. Always. | | | | | |
| Do you have sexual difficulties when you have sex with your partner, but not when you masturbate alone? Never. Almost never. Sometimes. Often. Almost always. Always. | | | | | |
| If it were up to you, could you forego sexual relationships?  Never. Almost never. Sometimes. Often. Almost always. Always. | | | | | |
| Do you feel depressed and nervous because you have sexual problems?  Never. Almost never. Sometimes. Often. Almost always. Always. | | | | | |
| If you have sexual problems, did they begin after: Illness, Pregnancy, Children, Contraceptive use, Medications, Toxic habits, Domestic violence, Relationship problems, Atopic dermatitis, Chronic illness, Which? | | | | | |
| What grade would you give your partner from 0 to 10? | | | | | |
| Do you have any comments? | | | | | |
| **DESIRE (Last 4 weeks)** | | | | | |
| How often did you feel sexual desire or interest?  Always or almost always / Most of the time / Sometimes. Rarely / Almost never or never. | | | | | |
| How do you rate your level (intensity) of sexual desire or interest?  Very high / High / Moderate / Low / Very low or nothing. | | | | | |
| How often did you feel sexual arousal during sexual activity?  I don't have sexual activity/ Always or almost always/ Sometimes/ Rarely/ Almost never or never | | | | | |
| How do you rate your level of sexual arousal during sexual activity?  I have no sexual activity / Very high / High / Moderate / Low / Very low or not at all. | | | | | |
| How confident are you that you will become aroused during sexual activity?  I am not sexually active / Very high confidence / Moderate confidence / Low confidence / Very low or no confidence | | | | | |
| How often were you satisfied with your arousal during sexual activity?  I don't have sexual activity/ Always or almost always/ Sometimes/ Rarely/ Almost never or never | | | | | |
| Do you find it difficult to get aroused during sexual activity (lubrication, erection)?  I am not sexually active/ Extremely difficult or impossible / Very difficult / Difficult / Somewhat difficult / Not difficult for me. | | | | | |
| How often do you reach orgasm or climax?  I don't have sexual activity/ Always or almost always/ Sometimes/ Rarely/ Almost never or never | | | | | |
| How satisfied are you with the emotional closeness that exists during sexual activity?  I am not sexually active / Very satisfied / Neither satisfied nor dissatisfied / Moderately dissatisfied / Very dissatisfied. | | | | | |
| How satisfied are you with your sexual relationship with your partner? Not sexually active / Very satisfied / Neither satisfied nor dissatisfied / Moderately dissatisfied / Very dissatisfied. | | | | | |
| How satisfied are you with your sex life overall? Not sexually active / Very satisfied / Neither satisfied nor dissatisfied / Moderately dissatisfied / Very dissatisfied. | | | | | |
| **ATOPIC DERMATITIS** | | | | | |
| F | Has it made it difficult to establish a relationship? | | | | |
| F | Has it prevented you from having your first sexual relationship? YES. NO. | | | | |
| F | Has it delayed your first sexual relationship? YES. NO. | | | | |
| F | Has it delayed your first masturbation? YES. NO. I've never masturbated. | | | | |
| F | In the last 4 weeks, has it prevented you from having sexual activity? | | | | |
| D | In the last 4 weeks, has your desire or interest in sex decreased? | | | | |
| D | Has your interest in having or maintaining a relationship decreased? YES. NO. | | | | |
| E | In the last 4 weeks, has this affected your arousal during sex? | | | | |
| S | In the last 4 weeks, has this affected your satisfaction during sexual intercourse? | | | | |
| S | In the last 4 weeks, have you experienced pain during sexual intercourse due to atopic dermatitis? | | | | |
| S | In the last 4 weeks, has it prevented you from reaching climax or orgasm? | | | | |
| **DESIRE** | | **EXCITION** | | | **SATISFACTION** |
| **ACTIVITY FREQUENCY** | |  | | |  |
